# A systematic intervention for assistance of patients with asthma in Brazil: partnership between university and public health system

**DOI:** 10.1186/1939-4551-8-S1-A211

**Published:** 2015-04-08

**Authors:** Priscila Palhas, Janaina M Melo, Virginia PL Ferriani, Ana Carla S Araujo, Persio Roxo Jr, Élcio Vianna, Marcos Borges, Adriana S Moreno, Luane M Mello, Rosa Ferreira, Marcos R Gonçalves, Jorgete M Silva, Andrea Cetlin, Rosangela Villela, Patricia Stefanelli

**Affiliations:** 1 Ribeirao Preto Medical School, University of Sao Paulo, Brazil; 2 Clinical Hospital of Ribeirao Preto Medical School, Brazil

## Background

Current GINA guidelines emphasize the challenge of asthma care programs to meet local needs in various countries. We aimed to asses outcomes of a one-year capacitating program on asthma for non-specialists working in the Public Health System in Brazil.

## Methods

A group of 16 allergists/immunologists developed a capacitating program in 11 Public Health Units in the city of Ribeirão Preto, Brazil. The program comprised lectures on asthma and hands-on training on spirometry and use of inhalation devices; production of didactic material; and development of a protocol on management of asthma. Spacers and spirometry were provided. Each researcher visited one Health Unit 2-4 times a month, to accompany the non-specialist on patients' visits to the clinic, perform case discussions, and deliver short lectures to the health professionals. Records of asthma medications provided to patients upon physicians' prescription in the North Region were compared to those from three other Regions with no intervention.

## Results

During the year prior to the capacitation program, inhaled Beclomethasone was delivered to 2,876 patients upon physician's prescription. At the end of the one-year capacitating program, there was an increase in deliver of inhaled Beclomethasone to 3,526 patients. Similarly, inhaled Albuterol, recommended as the rescue medication on the protocol, was delivered to 3,205 patients upon physician's prescription on the prior year, with increase at the end of the program to 4,850 patients. No such increase was observed in the South, Central and East Regions, where no capacitating program was conducted. Comparable number of prescriptions filled for inhaled Beclomethasone in the periods analyzed was: 1,210 and 1,074 for the East Region; 1,882 and 1,468 for the Central Region; and 1,347 and 1,244 for the South region, respectively. For inhaled Albuterol, the numbers were: 1,736 and 1,813 for the East; 2,602 and 2,662 for the Central; and 1,535 and 1,795 for the South Region, respectively. The number of prescriptions filled for Albuterol tablets and Aminophyline tablets decreased in most regions, whereas prescriptions filled for Albuterol syrup remained unchanged, with high prescription rates during the fall and winter months.

## Conclusions

A systematic capacitating program was successful in changing asthma prescription profiles among non-specialist doctors, with increasing delivery of inhaled Albuterol and inhaled Beclomethasone to patients with asthma.

